# Association Between Posterior Ankle Soft Tissue Properties and Deep Squatting Ability After Ankle Fracture Surgery: A Cross-Sectional Study

**DOI:** 10.3390/jfmk11030252

**Published:** 2026-06-26

**Authors:** Hayato Miyasaka, Bungo Ebihara, Makoto Takahashi, Takashi Fukaya, Koichi Iwai, Shigeki Kubota, Hirotaka Mutsuzaki

**Affiliations:** 1 Department of Rehabilitation, Tsuchiura Kyodo General Hospital, 4-1-1 Otsuno, Tsuchiura 300-0028, Ibaraki, Japan; miyasaka1853h@yahoo.co.jp (H.M.); bun.hirakata@gmail.com (B.E.); 2 Graduate School of Health Sciences, Ibaraki Prefectural University of Health Sciences, 4669-2 Ami, Ami 300-0394, Ibaraki, Japan; 3 Department of Physical Therapy, Japan University of Health Sciences, 2-555 Hirasuka, Satte 340-0145, Saitama, Japan; mtakahashi606@gmail.com; 4 Department of Physical Therapy, Tsukuba International University, 6-8-33 Manabe, Tsuchiura 300-0051, Ibaraki, Japan; t-fukaya@tius.ac.jp; 5 Center for Humanities and Sciences, Ibaraki Prefectural University of Health Sciences, 4669-2 Ami, Ami 300-0394, Ibaraki, Japan; iwai@ipu.ac.jp; 6 Department of Occupational Therapy, Ibaraki Prefectural University of Health Sciences, 4669-2 Ami, Ami 300-0394, Ibaraki, Japan; kubotashi@ipu.ac.jp; 7 Center for Medical Science, Ibaraki Prefectural University of Health Sciences, 4669-2 Ami, Ami 300-0394, Ibaraki, Japan; 8 Department of Orthopedic Surgery, Ibaraki Prefectural University of Health Sciences, 4773 Ami, Ami 300-0331, Ibaraki, Japan

**Keywords:** echo intensity, elastography, shear modulus, stiffness

## Abstract

**Background**: Deep squatting is essential for daily activities and sports; however, it is often limited after ankle fracture surgery, and the contributions of posterior ankle soft tissues, including the soleus muscle (SOL), Achilles tendon (AT), flexor hallucis longus muscle (FHL), and Kager’s fat pad (KFP), to this limitation remain unclear. This study aimed to determine the relationship between posterior ankle soft tissue properties (including stiffness and echo intensity [EI]) and deep squatting ability after ankle fracture surgery. **Methods**: This cross-sectional study included 53 patients (49.5 ± 16.1 years, 26 men) who underwent ankle fracture surgery. We measured the shear modulus of the SOL and AT, and the EI of the FHL and Kager’s fat pad; ankle range of motion and strength were evaluated. Deep squatting ability was also assessed. Multiple regression and receiver operating characteristic (ROC) analyses were performed to identify predictors of squatting limitation and evaluate discriminative performance. **Results**: Participants with a deep squatting limitation showed a higher shear modulus in the SOL and AT and higher EI in the FHL compared with those without limitations. SOL and AT shear modulus and FHL EI were significant independent predictors of ankle dorsiflexion angle during deep squatting. ROC analysis showed good discriminative ability for SOL shear modulus and AT shear modulus and modest discriminative ability for FHL EI. **Conclusions**: Increased stiffness and EI of the SOL, AT, and FHL were associated with reduced deep squatting ability after ankle fracture surgery. Targeted assessment and interventions addressing these tissues may improve postoperative function.

## 1. Introduction

Ankle fractures are among the most common lower-limb fractures, accounting for approximately 9% of all fractures [1,2]. With an aging population and the increasing prevalence of osteoporosis, fragility-related ankle fractures in older adults have risen and are projected to triple by 2030 [2,3,4]. Consequently, the clinical demand for postoperative rehabilitation following ankle fractures is expected to grow.

After ankle fracture surgery, swelling, pain, and reduced soft tissue flexibility often make deep squatting difficult [5,6]. Deep squatting, which is operationally defined as descending to the maximal depth with heel contact and maximal knee flexion below hip level, is a fundamental movement used in daily activities [7,8], agricultural work [7,8], and sports [9], and limitations in this movement may hinder social reintegration after surgery. Moreover, greater ankle dorsiflexion during deep squatting is significantly positively associated with postoperative quality of life [10], highlighting the importance of restoring this movement as a key rehabilitation outcome. Several factors influence deep squatting ability, including ankle dorsiflexion range of motion (ROM) and dorsiflexion strength [11]. Although postoperative limitations in ankle dorsiflexion may arise from both intra-articular restrictions and alterations in extra-articular soft tissue properties, the collective contribution of posterior ankle soft tissues remains poorly understood. Ankle dorsiflexion ROM is negatively correlated with Achilles tendon (AT) stiffness [12]. In addition, the soleus muscle (SOL), which functions as a single-joint plantar flexor, may be biomechanically related to deep squatting because it limits dorsiflexion when the knee is flexed [13]. Alterations in the properties of the flexor hallucis longus (FHL) and Kager’s fat pad (KFP), including stiffness and echo intensity (EI), have also been reported to restrict ankle dorsiflexion [14]. However, despite these findings, no study has comprehensively and quantitatively examined posterior soft tissue factors and their relationship to deep squatting ability after ankle fracture surgery.

Recently, shear wave elastography (SWE) and EI have been increasingly used to assess soft tissue properties [15,16]. In both healthy individuals [17] and patients following ankle fracture surgery [16], SWE-based assessments of the SOL and AT have demonstrated high reliability. As the FHL and KFP are located in deep regions near bone, where ultrasound refraction, reflection, and diffraction are more likely to occur, EI is recommended for evaluating their properties. These imaging modalities provide an opportunity to elucidate the contribution of posterior soft tissue properties to limitations in deep squatting ability.

Therefore, in this study, we aimed to investigate the relationship between posterior ankle soft tissue properties (including stiffness and EI) and deep squatting ability after ankle fracture surgery. We hypothesized that greater stiffness and EI of the SOL, AT, and FHL would be associated with reduced deep squatting ability. Confirmation of this relationship may inform rehabilitation strategies aimed at improving deep squatting ability through targeted modulation of posterior soft tissue properties.

## 2. Materials and Methods

### 2.1. Participants

**Figure 1:**
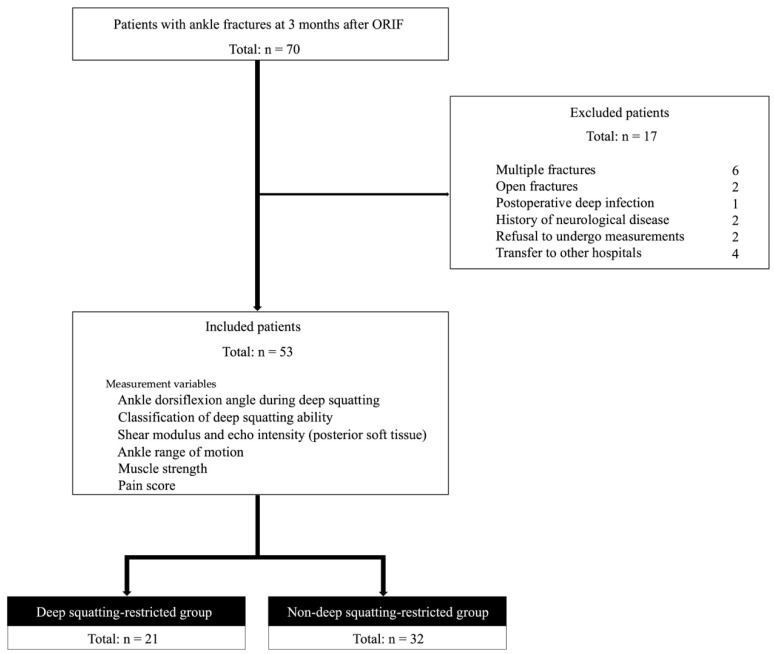
Flowchart of participant inclusion. Participants were classified according to their deep-squatting ability. Those who were unable to achieve the deep-squatting position owing to physical limitations were assigned to the restricted group, whereas those who successfully achieved the position were assigned to the non-restricted group.

The study was conducted between July 2022 and December 2025. We included patients with malleolar, bimalleolar, or tri-malleolar ankle fractures admitted to our hospital and undergoing open surgery and subsequent physical therapy. All patients underwent open reduction and internal fixation (ORIF) and were immobilized in a splint for at least 1 week after surgery. We excluded patients with multiple or open fractures, postoperative complications such as deep infection or deep vein thrombosis, and a history of neurological or orthopedic disorders and patients who were transferred to another hospital resulting in loss to follow-up (Figure 1).

Participants underwent measurements 3 months after ORIF. This time point was selected because it generally represents a transition period from postoperative immobilization and protected weight-bearing to more advanced functional recovery following ankle fracture surgery. All participants used crutches for at least the first 3 weeks and continued a standardized postoperative physical therapy program at our hospital for joint mobility, muscle strength, and functional skills, such as walking and stair climbing, for a minimum of 3 months. Participants were advised to maintain their usual daily activities but were instructed to avoid high-impact or strenuous physical activities outside the supervised rehabilitation program during the first 3 months after surgery. The participants’ age, sex, height, fracture type [18,19], and Lauge–Hansen classification [20] were collected from medical records. Participant weight was measured with a digital scale, and body mass index (BMI) was calculated.

### 2.2. Ankle Dorsiflexion During Deep Squatting

**Figure 2:**
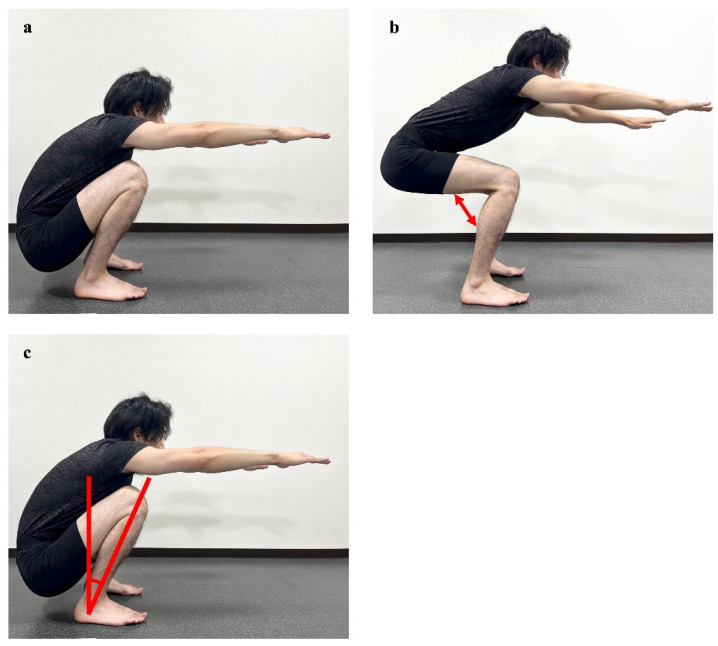
Deep squatting posture. (**a**) Lateral view of a participant without limitations in deep squatting ability. The hip, knee, and ankle joints are sufficiently flexed, allowing full contact between the thigh and lower leg while keeping the heels on the floor. (**b**) Lateral view of a participant with limitations in deep squatting ability. Due to reduced ankle dorsiflexion, the thigh and lower leg do not come into full contact while keeping the heels on the floor. The red arrow indicates the distance between the thigh and lower leg used to assess deep squatting ability. (**c**) Measurement of ankle dorsiflexion angle during deep squatting. The red line represents the angle between a line connecting the fibular head and lateral malleolus and a vertical line to the floor, which was used to quantify ankle dorsiflexion.

Deep squatting is defined as a posture in which the feet are placed shoulder-width apart, the arms are extended forward parallel to the floor, and the heels remain in contact with the ground while the thighs touch the calves [21]. Before measurement, participants received standardized verbal instructions and a demonstration of the movement. They were allowed one practice trial to familiarize themselves with the task. No specific warm-up protocol was provided; the measurement was performed during the routine outpatient assessment. Participants held this position for 3 s, during which ankle dorsiflexion was measured with a goniometer as the angle between a line connecting the fibular head and the lateral malleolus and a vertical line to the floor (Figure 2) [21]. The minimum recorded value was 1°. The intra-rater reliability of this measurement method is 0.986 (95% confidence interval, 0.973–0.993) [10]. Deep squatting ability was assessed by measuring the distance between the thigh and the lower leg in the deep squatting position. Full deep squatting has been described as a posture in which maximal knee flexion is achieved with the thighs contacting the calves [21]. Accordingly, a limitation in deep squatting ability was defined as a thigh–lower leg distance greater than 0 cm. Patients were classified into two groups based on this distance: a deep squatting-restricted group (>0 cm) and a non-deep squatting-restricted group (0 cm) (Figure 2). This classification was used for subsequent analyses of factors associated with deep squatting ability.

### 2.3. Shear Modulus and EI

All ultrasound assessments were conducted using a 2–10 MHz linear transducer (Supersonic Imaging, Aix-en-Provence, France). A physical therapist with 9 years of experience in musculoskeletal ultrasonography evaluated the shear modulus of the SOL muscle and AT using the SWE Opt penetration setting and measured the EI of the FHL and KFP in B-mode. Ultrasound images were consistently acquired along the longitudinal axis of the soft tissues. Participants were seated and rested for 10 min before the assessment and were instructed to remain fully relaxed throughout the procedure. All measurements were performed before the deep squatting procedure.

The SWE technique uses the acoustic radiation force from the push pulse to generate shear waves in individual tissues and calculates shear modulus using the following equation:

μ (kPa) = ρVs^2^,

where μ is the shear modulus, ρ is the tissue density (assumed to be 1000 kg/m^3^ for muscle), and Vs is the shear wave velocity.

**Figure 3:**
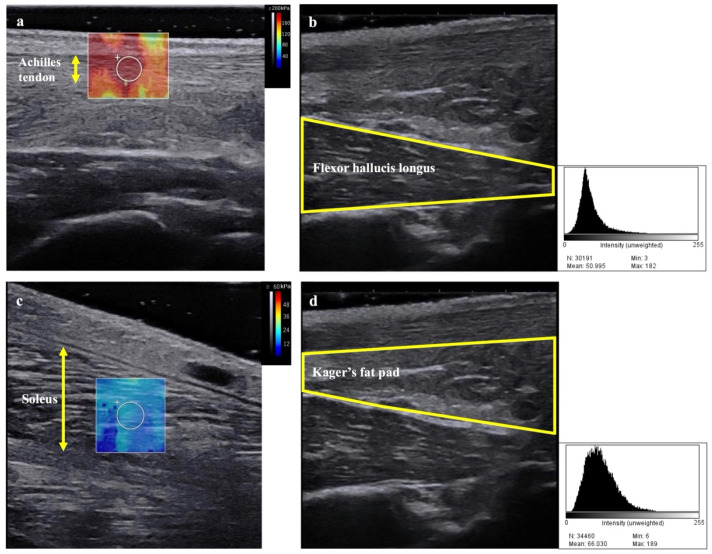
Illustration of the shear modulus and echo intensity measurements. (**a**) Shear wave elastography image of the Achilles tendon. (**b**) Echo intensity image of the flexor hallucis longus muscle. (**c**) Shear wave elastography image of the soleus muscle. (**d**) Echo intensity image of Kager’s fat pad. The circle indicates the region of interest, and the box indicates the analysis area.

Measurements were taken in a kneeling position with both knees flexed at 90°. The upper body was supported on a 45 cm table to ensure relaxation of the lower limbs. Participants were instructed to remain fully relaxed during measurements and to avoid exercise that could induce muscle soreness for 48 h prior. The ankle dorsiflexion angle was set to 10° using a goniometer, and the foot was secured to the footplate with a strap. The SOL was measured around the musculotendinous junction of the gastrocnemius, while the AT, FHL, and KFP were measured at a point 3 cm proximal to the calcaneal tuberosity. Measurement areas were identified using transverse images and marked on the skin with a black pen. Shear modulus ranges were set to 0–60 kPa and 0–200 kPa for the SOL and AT, respectively. The probe was placed parallel to the SOL and AT fibers and held stationary for 10 s using a region of interest (ROI) circle of 4 mm and 3 mm diameter, respectively. A large amount of gel was used, and the probe was applied without pressure to obtain good shear wave propagation. The room temperature was maintained at 25 °C to prevent changes in soft tissue stiffness due to temperature [22]. The mean value of shear modulus was adopted (Figure 3).

The EI was derived by converting the pixels of the stored B-mode images into 8-bit grayscale values (range: 0–255, from black to white) using ImageJ software version 1.54i (National Institutes of Health, Bethesda, MD, USA) [23]. For B-mode acquisition, the gain was maintained at 50%, and images were obtained only when both fascia and underlying bone were clearly delineated. The dynamic range was fixed at 72 dB, and the focal depth was adjusted between 2.0 and 4.0 cm depending on individual anatomical characteristics. For EI calculation, the ROI was maximized to fit the contour of the soft tissue while ensuring that fascia and bone structures were excluded. The mean EI value was used for analysis (Figure 3).

### 2.4. Ankle ROM

Ankle ROM was measured in 1° increments using a goniometer (MMI universal goniometer Todai 300 mm; Muranaka Medical Instruments, Co., Ltd., Osaka, Japan) during passive movement. Participants remained supine, ankle dorsiflexion ROM was measured with the knee in extended and flexed positions, and plantarflexion ROM was measured with the knee in the flexed position [24]. Passive ankle ROM was evaluated by manually guiding the participant’s foot to the maximal dorsiflexion and plantarflexion positions. The examiner set the axis perpendicular to the fibula and aligned the plantar surface of the foot as the axis of movement, and the angle between these axes was measured as ankle dorsiflexion and plantarflexion ROM.

### 2.5. Ankle Muscle Strength

Ankle muscle strength was assessed using a Biodex dynamometer (Biodex Medical Systems, Shirley, NY, USA) to evaluate plantarflexion and dorsiflexion capacity. Participants were seated with their knees flexed at 30°, and straps were applied to stabilize the lower trunk, thighs, and ankles. Isokinetic measurements of ankle plantarflexion and dorsiflexion were performed bilaterally in a concentric–concentric mode. Two sets of five maximal dynamic repetitions were conducted at an angular velocity of 60°/s, with a 30 s rest between sets [21]. Participants’ feet were kept parallel to the floor to minimize hamstring strain. Peak torque normalized to body weight was calculated from measurements exceeding 1 Nm.

### 2.6. Ankle Pain During Deep Squatting

Under standardized testing conditions, participants rated their ankle pain on a 100 mm visual analog scale [25] immediately after holding the deep squatting position for 3 s.

### 2.7. Statistical Analysis

The required sample size was estimated using G*Power 3.1 (Heinrich Heine University, Germany) [26]. Based on a multiple regression model with an assumed effect size of f^2^ = 0.35 for the outcome of ankle dorsiflexion during deep squatting, a significance level of 0.05, and a statistical power of 0.80, the analysis indicated that at least 51 participants were necessary. Accordingly, 53 individuals were included in the present study.

Data distribution was examined using the Shapiro–Wilk test. Variables following a normal distribution are expressed as mean ± standard deviation, whereas non-normally distributed variables are reported as median with interquartile ranges. Group differences between participants with and without deep squatting limitations were analyzed using either independent *t*-tests or Mann–Whitney U tests, selected according to the distribution of each variable. Sex and fracture type differences between groups were evaluated using the chi-square test or Fisher’s exact test when cell counts were small. Effect sizes (r) were calculated for all group comparisons from the relevant test statistics and reported as standardized measures of effect magnitude. To determine factors influencing ankle dorsiflexion angle during deep squatting, simple linear regression and multiple regression analyses using forced-entry methods were performed, incorporating AT and SOL shear modulus and EI values of the FHL and KFP as candidate predictors, with adjustment for age, BMI, and fracture type. Receiver operating characteristic (ROC) analyses [27] were conducted, not to establish diagnostic thresholds but to compare how well the AT and SOL shear modulus and FHL EI values differentiated the predefined groups with and without deep squatting restriction. Discriminative performance was quantified using the area under the curve (AUC), sensitivity, specificity, and optimal cutoff values derived from the Youden index.

A significance level of *p* < 0.05 was applied throughout. All analyses were completed using SPSS Statistics version 31 (IBM Corp., Armonk, NY, USA). No missing data were present in the analyzed dataset.

## 3. Results

### 3.1. Characteristics of Participants with and Without Deep Squatting Limitation

Figure 1 illustrates the participant selection process. A total of 70 patients underwent surgical treatment for ankle fractures at our institution. Seventeen patients were excluded due to multiple fractures (n = 6), open fractures (n = 2), postoperative deep infection (n = 1), a history of neurological disease (n = 2), refusal to participate in the measurements (n = 2), or transfer to another hospital (n = 4). Consequently, 53 patients were included in the final analysis. The participants’ characteristics with and without deep squatting limitation are summarized in Table 1. The time from ORIF to measurement was 92.4 ± 5.9 days.

### 3.2. Comparison Based on Deep Squatting Ability

Table 2 presents a comparison of the outcome measures based on the presence or absence of deep squatting limitation. The restricted group showed a significantly smaller ankle dorsiflexion angle during deep squatting (18.0 ± 2.3 vs. 26.7 ± 3.8°, *p* < 0.001, effect size r = 0.84), as well as significantly higher shear modulus values in the SOL (24.1 ± 3.7 vs. 19.9 ± 3.1 kPa, *p* < 0.001, effect size r = 0.54) and AT (209.1 ± 19.7 vs. 188.0 ± 15.7 kPa, *p* < 0.001, effect size r = 0.52), and higher EI values in the FHL (47.3 ± 27.1 vs. 31.7 ± 17.8 a.u., *p* = 0.026, effect size r = 0.38) than the non-restricted group.

### 3.3. Linear Regression Analyses

The simple linear regression analysis revealed that the shear modulus of the SOL (β = −0.689, *p* < 0.001) and of the AT (β = −0.631, *p* < 0.001), and EI of the FHL (β = −0.401, *p* = 0.003) were all significantly associated with ankle dorsiflexion angle in the deep squatting position. In the multiple linear regression model, the shear modulus of the SOL (β = −0.405, *p* = 0.003) and AT (β = −0.326, *p* = 0.012), and EI of the FHL (β = −0.203, *p* = 0.049) remained independent determinants of the dorsiflexion angle (Table 3). The multiple correlation coefficient (R) and adjusted coefficient of determination (R^2^) were 0.752 and 0.539, respectively. The Durbin–Watson statistic was equal to 1.252, indicating no meaningful autocorrelation in the residuals (*p* = 0.86).

### 3.4. Deep Squatting Ability Prediction

**Figure 4:**
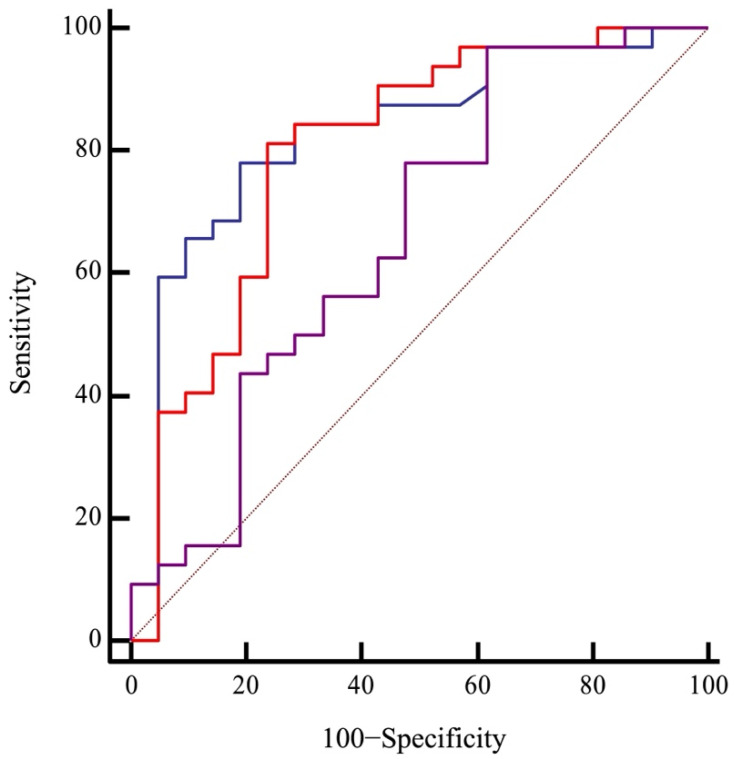
Receiver operating characteristic curves for the shear modulus of the SOL, shear modulus of the AT, and EI of the FHL in discriminating deep squatting ability. The blue, red, and purple lines represent the shear modulus of the SOL, shear modulus of the AT, and EI of the FHL, respectively.

The ROC curve analysis for the shear modulus of the SOL (Figure 4) demonstrated an AUC of 0.83 (95% CI, 0.71–0.95). At a cutoff value of 21.3 kPa, the model demonstrated a sensitivity of 78.1% and a specificity of 81.0%. The ROC curve analysis for the shear modulus of the AT (Figure 4) demonstrated an AUC of 0.80 (95% CI, 0.71–0.95). At a cutoff value of 200.0 kPa, the model demonstrated a sensitivity of 81.2% and a specificity of 76.2%. In the ROC curve for the EI of the FHL (Figure 4), the AUC was 0.66 (95% CI, 0.50–0.82). Using a cutoff value of 57.2 a.u., the sensitivity and specificity were 96.9% and 38.1%, respectively.

## 4. Discussion

We examined the relationship between the properties of posterior ankle soft tissues, including stiffness and EI, and the ability to perform deep squatting after ankle fracture surgery. The results supported our hypothesis that greater stiffness and EI of the SOL, the AT, and the FHL are significantly associated with reduced deep squatting ability. These findings suggest that quantitative assessment of posterior ankle tissue properties, particularly of the SOL, AT, and FHL, may assist clinicians in identifying patients at risk of limited deep squatting ability after ankle fracture surgery and may guide individualized rehabilitation planning.

The SOL is a single-joint plantar flexor that contributes to ankle dorsiflexion ROM when the knee is flexed [13]. In the postoperative management of ankle fractures, immobilization with a cast or splint is common, and the ankle is frequently maintained in a plantarflexed or neutral position with restricted loading [28], which may promote increased SOL stiffness. As deep squatting requires substantial ankle dorsiflexion with the knee flexed [29], stiffness of the SOL is likely to limit this movement. The FHL is anatomically positioned posterior to the talus [14]. During normal dorsiflexion, the talus glides posteriorly to achieve full ROM [30]. Fibrotic changes around the FHL may restrict this posterior glide, contributing to dorsiflexion limitations. After ankle fracture surgery, fibrosis can develop due to posterior malleolar fracture involvement near the FHL or peripheral ankle inflammation. These mechanisms provide a plausible explanation for the observed association between increased FHL EI and limited deep squatting. Studies in healthy individuals have examined factors related to deep squatting, focusing on single structures such as the AT [21] or the triceps surae [31]. For example, greater AT stiffness has been associated with reduced ankle dorsiflexion at maximum squat depth [21], and increased SOL shear modulus has been reported to restrict deep squatting motion during increased ankle dorsiflexion [31]. In contrast, our study provides a more comprehensive evaluation by assessing the contributions of multiple posterior soft tissues in patients who experienced deep squatting limitations after ankle fracture surgery. By quantifying site-specific muscle stiffness using ultrasound imaging, this study adds new insights to the current understanding of posterior soft tissue contributions. Our findings suggest that evaluating the properties of the SOL, the AT, and the FHL may offer more clinically relevant information than assessments limited to the KFP. In the present study, the KFP was analyzed as a single structure, despite previous reports [32] suggesting that it consists of multiple functional and anatomical subregions. This simplification was primarily due to the difficulty of consistently differentiating these subregions using dynamic ultrasound imaging. As a result, the ROI may have encompassed both portions adjacent to the AT and those related to the FHL. Such an approach may have obscured potential regional differences in echogenicity and functional relevance, thereby reducing the sensitivity of KFP measurements in detecting associations with deep squatting ability. These results support the need for targeted, site-specific evaluations in patients following ankle fracture surgery when assessing complex functional tasks such as deep squatting. Future studies should explore methods to evaluate anatomically distinct regions of the KFP separately, as regional differences may have functional relevance to ankle dorsiflexion and deep squatting ability.

ROC curve analyses were conducted to evaluate the ability of the SOL shear modulus, AT shear modulus, and FHL EI to differentiate individuals with and without deep squatting limitations, with diagnostic accuracy assessed by the AUC, sensitivity, and specificity. The shear modulus of the SOL demonstrated good diagnostic performance, with an AUC of 0.83. At a cutoff value of 21.3 kPa, the model achieved a sensitivity of 78.1% and a specificity of 81.0%. The shear modulus of the AT also demonstrated good diagnostic performance, with an AUC of 0.80. At a cutoff value of 200.0 kPa, the model achieved a sensitivity of 81.2% and a specificity of 76.2%. The EI of the FHL showed modest discriminative capability, with an AUC of 0.66, a sensitivity of 96.9%, and a specificity of 38.1% at a cutoff value of 57.2 a.u. This result suggests that the FHL EI may be more useful for screening purposes due to its high sensitivity, despite its relatively low specificity. Clinically, these findings raise the possibility that interventions aimed at reducing stiffness in the SOL and AT as well as addressing FHL tissue properties could aid in restoring deep squatting ability. For example, patients exceeding the identified cutoff values for SOL and AT shear modulus may be at a higher risk of restricted ankle dorsiflexion during functional tasks and could benefit from targeted interventions aimed at reducing muscle-tendon stiffness, including stretching or mobility-focused exercises. In contrast, the high sensitivity of FHL EI suggests that it may serve as a useful screening tool to identify patients who are at risk of not achieving full deep squatting ability, although its lower specificity limits its utility for definitive decision-making. Collectively, these measures may assist clinicians in prioritizing assessment of specific posterior soft tissues and tailoring rehabilitation strategies according to individual tissue characteristics. Although some studies in healthy young adults have shown that static stretching combined with whole-body vibration can decrease SOL and AT stiffness [33], the applicability of this approach to patients after ankle fracture surgery remains unclear. Evidence regarding interventions specifically targeting FHL tissue properties is also lacking. Therefore, additional investigations are needed to determine treatment strategies that are both effective and safe for clinical application.

This study has some limitations. First, although the sample size was determined based on a power analysis, it remained relatively small and was acquired from a single institution. As a result, the generalizability of the findings may be limited when applied to broader patient populations with varying fracture patterns, surgical techniques, and rehabilitation protocols. Second, because of the cross-sectional design and the assessment of posterior soft tissue properties and deep squatting ability at a single postoperative time point, temporal changes and causal relationships between posterior soft tissue properties and deep squatting ability could not be determined. Longitudinal studies are needed to clarify these relationships over time. Third, the lack of a control group limits interpretation of the extent to which the observed soft tissue properties are specific to patients after ankle fracture surgery. Fourth, although all participants were evaluated at approximately 3 months after surgery, heterogeneity in age, fracture type, and other participant characteristics may have introduced bias, and stratified analyses were not performed. Fifth, hardware-related factors (such as the presence of plates and screws after ORIF) may mechanically restrict posterior ankle soft tissue mobility and potentially influence ultrasound-based measurements, including SWE and EI, by altering tissue deformation, sliding behavior, and image acquisition conditions. The hardware may also have affected SWE measurements of the AT and SOL; additionally, implant-related variables, including implant type, location, and prominence, were not assessed in this study. Therefore, their potential confounding effects cannot be excluded. Future studies should consider these factors when interpreting postoperative soft tissue properties. Finally, the assessed properties (stiffness and EI) of the SOL, AT, and FHL may capture only a portion of each muscle’s overall mechanical characteristics and quality, indicating that our results may not fully reflect their complete functional status. However, the measurement sites selected in this study correspond to clinically relevant regions that are most likely to influence deep squatting performance and postoperative ankle function. Future studies should include larger, multicenter cohorts; longitudinal assessments; appropriate control groups; stratified analyses based on participant characteristics and fracture type; and comprehensive evaluations of the entire muscle architecture to further validate these findings and guide targeted rehabilitation strategies.

## 5. Conclusions

In conclusion, greater stiffness and EI of the SOL, AT, and FHL is associated with reduced deep squatting ability after ankle fracture surgery. Targeted assessment and interventions addressing these posterior soft tissues may contribute to functional recovery. Future studies should validate these findings in larger, multicenter cohorts and investigate whether interventions aimed at modifying the mechanical properties of these tissues, such as stretching or soft-tissue-focused treatments, can improve deep squatting ability and functional recovery.

## Figures and Tables

**Table 1:** Participant characteristics with and without deep squatting limitation.

Variables	Deep Squatting Ability		*p* Value	Effect Size
	Restricted Group (n = 21)	Non-Restricted Group (n = 32)		
Age (years)	54.4 ± 17.7	44.9 ± 22.1	0.167	0.19
Sex (male/female)	8/13	18/14	0.196	0.18
Height (m)	1.61 ± 0.10	1.62 ± 0.08	0.978	0.01
Weight (kg)	64.5 (59.0–75.1)	56.3 (50.0–70.5)	0.025	0.31
Body mass index (kg/m^2^)	26.7 ± 4.6	23.0 ± 4.2	0.004	0.38
Fracture type (malleolar/bimalleolar/trimalleolar)	6/3/12	17/6/9	0.100	0.20

**Table 2:** Comparison of deep squatting ability between the restricted and non-restricted groups.

Variables	Deep Squatting Ability		*p* Value	Effect Size
	Restricted Group (n = 21)	Non-Restricted Group (n = 32)		
Shear modulus (kPa)				
Achilles tendon	209.1 ± 19.7	188.0 ± 15.7	<0.001	0.52
Soleus muscle	24.1 ± 3.7	19.9 ± 3.1	<0.001	0.54
Echo intensity (a.u.)				
Flexor hallucis longus	47.3 ± 27.1	31.7 ± 17.8	0.026	0.38
Kager’s fat pad	60.3 ± 31.3	50.5 ± 23.3	0.225	0.21
Dorsiflexion ROM with knee extended (°)	11.0 ± 2.4	13.8 ± 2.4	<0.001	0.49
Dorsiflexion ROM with knee flexed (°)	13.4 ± 2.6	18.2 ± 3.1	<0.001	0.62
Plantarflexion ROM (°)	56.0 ± 5.8	59.4 ± 4.8	0.056	0.26
Dorsiflexion angle during deep squatting (°)	18.0 ± 2.3	26.7 ± 3.8	<0.001	0.84
Plantarflexion muscle strength (Nm/kg)	0.26 ± 0.13	0.45 ± 0.19	<0.001	0.48
Dorsiflexion muscle strength (Nm/kg)	0.28 (0.21–0.31)	0.35 (0.26–0.42)	0.024	0.31
Ankle pain during deep squatting (mm)	26.1 ± 17.8	13.1 ± 17.3	0.008	0.36

Groups were defined based on participants′ clinical ability to assume the deep squatting at 3 months postoperatively. Restricted: unable to assume the deep squatting posture; non-restricted: able to assume the deep squatting posture. ROM, range of motion.

**Table 3:** Multiple linear regression analyses.

Independent Variables	Dependent Variables	Multiple Linear Regression							
		B	95% CI	β	SE	*p* Value	R^2^	f^2^	VIF
Dorsiflexion angle during deep squatting	SOL shear modulus	−0.562	−0.928 to −0.197	−0.405	0.182	0.003	0.539	1.299	1.931
	AT shear modulus	−0.088	−0.156 to −0.020	−0.326	0.034	0.012			1.776
	FHL EI	−0.048	−0.095 to −0.001	−0.203	0.024	0.049			1.130
	KFP EI	−0.037	−0.192 to 0.104	−0.183	0.033	0.274			1.109

AT, Achilles tendon; B, partial regression coefficient; CI, confidence interval; FHL EI, flexor hallucis longus echo intensity; KFP EI, Kager’s fat pad echo intensity; β, standardized regression coefficient; R^2^, adjusted coefficients of determination; f^2^, effect size; VIF, variance inflation factor; SE, standard error; SOL, soleus muscle.

## Data Availability

The data supporting the findings of this study are available upon request from the corresponding author. The data are not publicly available owing to restrictions on their containing information that could compromise the privacy of the research participants.

## Abbreviations

The following abbreviations are used in this manuscript:

ROM	range of motion
AT	Achilles tendon
SOL	soleus muscle
FHL	flexor hallucis longus
KFP	Kager’s fat pad
EI	echo intensity
SWE	shear wave elastography
ORIF	open reduction and internal fixation
BMI	body mass index
ROI	region of interest
ROC	receiver operating characteristic
AUC	area under the curve
